# Current Status for Gastrointestinal Nematode Diagnosis in Small Ruminants: Where Are We and Where Are We Going?

**DOI:** 10.1155/2014/210350

**Published:** 2014-09-02

**Authors:** Sarah Jane Margaret Preston, Mark Sandeman, Jorge Gonzalez, David Piedrafita

**Affiliations:** ^1^Biotechnology Research Laboratories, School of Biomedical Sciences, Monash University, Melbourne, VIC 3800, Australia; ^2^Faculty of Science, Federation University, Northways Road, Churchill, VIC 3841, Australia; ^3^Facultad de Veterinaria, Universidad de Las Palmas de Gran Canaria, Trasmontaña s/n, Arucas, Las Palmas 35413, Spain; ^4^School of Applied Sciences & Engineering, Faculty of Science, Gippsland, Northways Road, Churchill, VIC 3841, Australia

## Abstract

Gastrointestinal nematode (GIN) parasites pose a significant economic burden particularly in small ruminant production systems. Anthelmintic resistance is a serious concern to the effective control of GIN parasites and has fuelled the focus to design and promote sustainable control of practices of parasite control. Many facets of sustainable GIN parasite control programs rely on the ability to diagnose infection both qualitatively and quantitatively. Diagnostics are required to determine anthelmintic efficacies, for targeted treatment programs and selection of animals for parasite resistant breeding. This review describes much of the research investigated to date to improve the current diagnostic for the above practices which is based on counting the number of parasite eggs in faeces.

## 1. Introduction

Small ruminant (goats and sheep) production systems worldwide are significantly constrained by gastrointestinal nematode (GIN) parasites, reducing meat, milk, and fibre production [1–3]. Anthelmintic treatment is the most cost-effective current control method in many farm enterprises. However, the ability of parasites to quickly develop resistance to these compounds, particularly if animals are under-dosed or treated under preventative and suppressive treatment regimes, suggests alternative and/or complementary sustainable control programs require adoption [4–8].

Sustainable control programs and guidelines (Table 1) have been introduced to small ruminant producers to prolong the effectiveness of anthelmintics whilst reducing the production loss caused by GIN parasite infections (reviewed by [9–13]). These programs/guidelines involve a combination of chemical and nonchemical strategies to adequately control GIN parasites; however, the success or otherwise of these programs is reliant on an ability to diagnose the parasitic infections qualitatively and quantitatively to estimate the severity of the infection and the potential cost to production traits [14, 15].

Alternatives and/or complementary solutions to anthelmintic use are also under investigation with breeding for genetic resistance of the host to infection already in commercial application (http://www.nsip.org; http://www.sheepgenetics.org.au/; http://www.signetfbc.co.uk/). Diagnostic assays that reliably measure the level of genetic resistance are required for such applications and a range of indicators have been or are being investigated and will be reviewed below.

## 2. Ideal Characteristics of Diagnostic Markers for GIN Parasite Infections

The ideal diagnostic test for GIN parasite infections has been described as having the following characteristics [14, 16]:

1. reliability in terms of accuracy and repeatability
2. ease of measurement
3. cost effectiveness
4. the ability to be used on-farm.

Additional characteristics for diagnostics that could be used in programs aimed at breeding for resistance include

(5) neutral or positive correlations with production traits,
(6) moderate to high heritability.

Although these parameters are easily defined, in practice, there is no universal marker currently that meets these characteristics for GIN parasite diagnosis. The most common test in use currently is the Worm Egg Count (WEC). The WEC test is promoted by government and nongovernment agencies in many commercialised small ruminant industries (Table 1). WEC has many technical disadvantages (Section 2.1) and poor adoption rates by farmers and is, therefore, considered an underutilised diagnostic in many small ruminant production systems [17]. As such, substantial research is being undertaken to find a more usable and accurate measure of infection intensity to assist the development and adoption of sustainable GIN parasite control programs. This review will examine diagnostic markers currently in use and those that are being considered or are currently under development with the potential to replace/improve WEC as a diagnostic marker. These diagnostic markers often involve components of the animals immune system and can be categorised under three major areas: infection-related, immune-related, and inherent markers.

### 2.1. Infection-Related Diagnostic Markers of GIN Parasitic Infection

Infection-related markers have been characterised by their dependence on current infection and are related to parasite induced pathology such as blood loss or a measure of parasite burden such as number of eggs.  Table 2 summarises the markers associated with infection investigated to date.

#### 2.1.1. Worm Egg Counts

WEC involves counting parasitic eggs in freshly collected faeces. Distinctive eggs from GIN parasites such as thin-neck intestinal worm (*Nematodirus *spp.), tape worm (*Moniezia expansa*), and whip worm (*Trichuris ovis*) can be easily identified. However, the worm species that have been identified to cause the largest economic impact on small ruminant production:* Trichostrongylus *spp.*, Haemonchus contortus*, and* Teladorsagia circumcincta* are difficult to distinguish by egg morphology and, therefore, require further processing. Identification to at least genus level is important for correct anthelmintic selection but less important for the use of diagnostics as a selection marker in breeding the host for resistance because resistance is usually expressed to a range of parasite species [18–20].

Previously, identification of eggs to species level has involved larval culture requiring at least 7–10 days for egg hatch; however, recently, polymerase chain reaction (PCR) techniques have transformed testing, allowing species identification in less than 24 hours [21–23]. This is an exciting development particularly its potential to identify GIN parasite resistant to anthelmintic classes directly from the faeces as farmers perceive the current protocol to detect anthelmintic resistance involving mini-sheep trials as too difficult and time-consuming [17].

Other research involved in transforming WEC diagnostic into a more time and cost efficient method is to use fluorescently labelled lectins which bind differentially to different GIN parasite species eggs [24–26]. A lectin test for the identification of* H. contortus* eggs is now commercially available through the Australian Government (http://www.sheepcrc.org.au/management/worms-flies-lices/rapid-laboratory-test-for-haemonchus-in-worm-egg-counts.php). Both improvements (DNA technology and lectin staining) do not overcome the collection of faeces and both must be performed off-farm by laboratory experts; however, there are reports for the potential of some DNA-technology platforms to become on-farm diagnostics in the future [27].

Other drawbacks of WEC tests commonly cited in the literature include the low correlation between eggs and worm burden for low fecund worms such as for* T. circumcincta* and* T. colubriformis, *inability to detect worms in hypobiosis, and the high variability of eggs between individual subsamples due to aggregation [15, 28]. In addition, collection of faeces is time and labour intensive and often an unpalatable technique for many farmers and animal health professionals. Despite these disadvantages, WEC tests are promoted commercially as a diagnostic tool to determine if anthelmintics are still effective on farming properties, for targeted anthelmintic treatment strategies and for the selection of animals for breeding parasite resistance through estimated breeding values in many countries [15].

#### 2.1.2. Blood Loss

Measurement of blood loss can indicate the presence of infection with a blood-feeding GIN parasite, such as* H. contortus*, and this parameter has been utilised as a diagnostic tool to target animals for anthelmintic treatment and selection of parasite resistant sheep. Tools available to measure blood loss include packed cell volume (PCV), the Haemonchus dipstick, and FAMACHA^©^. PCV is an indicator of anaemia and is usually used in conjunction with WEC to diagnose* H. contortus* infections for research purposes. PCV involves taking blood samples and measuring the percentage of red blood cells. The measurement of blood loss has an advantage over WEC in that it enables early detection of* H. contortus* infection as blood loss occurs prior to egg production [15]. However, blood sampling is labour intensive for farmers and blood loss can be due to infections other than* H. contortus* such as a severe coccidial or bacterial enteritis infections [14, 29].

To overcome the labour-intensive nature of measuring PCV, the Australian Government commercialised a product, the Haemonchus Dipstick, which measures blood loss by quantifying the amount of blood in the faeces [29]. Although this is a much more practical way of measuring blood loss for producers and can identify infection earlier than WEC to prevent sudden disease outbreaks in high risk periods [11], the concern of the exact origin for the blood loss remains [29]. Therefore, a WEC test is recommended to be used to confirm that blood loss is due to* H. contortus* infection and not another underlying physiological problem or infection [29].

FAMACHA^©^ is a* H. contortus*-specific diagnostic tool developed in South Africa, which uses the colour of the eye as an indicator of anaemia due to the parasite's blood feeding activity [30–32]. FAMACHA^©^ is a five-point scoring system in which goats or sheep scored at 3–5 are deemed at risk of disease and require treatment [30]. Trials using FAMACHA^©^ as a selection tool in targeted selection anthelmintic treatment programs have shown reductions in the number of anthelmintic treatments with minimal production losses [31–33]. The major drawback of this tool as with the Haemonchus Dipstick is that anaemia is not exclusively caused by* H. contortus* infections. Despite this, a recent report describes high adoption rates in the southern states of the United States, with 5,000 small ruminant producers being trained through workshops to diagnose anaemic sheep based on FAMACHA^©^ and the purchase of 20,000 FAMACHA^©^ cards (reviewed by [13]).

FAMACHA^©^ can also be applied as a tool to breed* H. contortus *resistant sheep. Trials in United States and South Africa have shown moderate heritability values similar to WEC and PCV and positive associations with increased production traits [34, 35]. The relative ease and low cost of the FAMACHA^©^ system are advantageous for implementation, but it is only effective in areas where livestock are dominantly infected by* H. contortus,* and, as outlined above for the Haemonchus Dipstick, it lacks specificity.

#### 2.1.3. Faecal Odour

Only one research group has investigated the use of odour to detect GIN parasite infections, by training canines to detect the scent [36]. The limited research in odour detection of GIN parasites is surprising, given anecdotal evidence of producers being able to smell GIN parasite infections, in combination with the routine use of canines to detect explosives, illegal drugs, and human remains and detection of humans with ill-health (reviewed by [37, 38]). The work indicated that this method for detecting GIN parasite infection has high sensitivity, detecting* T. circumcincta* infections in sheep as early as seven days post oral infection with an 85% accuracy [36], potentially meaning that the odour diagnosis of GIN parasite infections could occur before egg laying and before clinical symptoms appear. Richards et al. [36] suggested that further work should focus on defining the chemical composition of the detectable odour to transform this knowledge into a detection device. The potential of this device to operate on-farm is promising, however, whether level of odour correlates strongly with infection level, a prerequisite for likely commercial success, requires investigation.

#### 2.1.4. Animal Behaviour

Parasite infections influence animal behaviour and some studies have been conducted to determine whether changes in behaviour would allow identification of parasite resistant or susceptible animals [39–41]. A recent study using a global positioning system (GPS) tracking device to monitor the behaviour of sheep under natural field infection conditions found that animals with higher WEC (more susceptible) travelled significantly greater distances than animals with lower WEC [42]. Theories as to why animals with heavier infections travelled greater distances included that these animals need to graze for longer periods to cope with protein loss due to infection and visit water sources more frequently due to an increased thirst [42]. GPS devices are still too expensive to be considered as a commercial tool for diagnosing GIN parasite resistant sheep, but they are an excellent research tool and further studies would be of interest.

#### 2.1.5. Weight Loss

The Happy Factor is a performance based marker which involves calculation of body weight gain based on food efficiency rates [43]. Animals which do not reach the predicted target are treated with anthelmintics. An advantage of using body weight scores is that condition loss is an early symptom of infection and is an economically important trait [43]. A recent study based in a commercial setting in the United Kingdom showed that this approach resulted in a 50% decrease in anthelmintic treatment [44]. However, animals needed to be weighed fortnightly, which the authors acknowledge may not be practical in all livestock production settings.

#### 2.1.6. Worm Number and Weight

Worm number and weight is the most direct measurement of determining GIN parasite infection levels and is consequently considered the gold standard for estimating parasitic worm burden [14]. Worm number involves counting the parasites in the gastrointestinal tract; a proportion of the worm population is usually measured as an estimate of the total [45]. Infective larval stages (L3, L4) and adult female and male GIN parasites can be enumerated and differentiated by morphology providing important information on the target of host resistance and are required for testing the activity of new anthelmintics [14]. Measuring worm weight involves collecting the parasites and recording their bulk weight. However, these markers can obviously only be taken at necropsy, are labour intensive, and are consequently only useful for research purposes.

### 2.2. Immune-Related Diagnostic Markers of GIN Parasitic Infection

It has long been established that the immune system plays a major role in resistance to GIN parasite infection [46, 47]. Immune cell depletion and cytokine profile studies have shown that resistance to infection is dependent on the induction of the type two (T2) or the “allergic” phenotype response [48–50]. The Th2 immune response is characterised by the differentiation of T cells that produce the cytokines IL-4, IL-5, IL-9, and IL-13, the proliferation and recruitment of effector cells, and eosinophils, mucosal mast cells and globular leukocytes, along with increased mucus secretion and generation of parasite-specific antibodies such as IgA, IgG_1_ and IgE [48, 51].

However, it is now recognised that manifestations before and after the T2 “allergic” immune response are also vitally important for successful control of infection [52]. Detailed studies in mice have shown the importance of correct innate receptor expression and functioning to recognise pathogen-associated molecular patterns and damage-associated molecular patterns (reviewed by [53]). Furthermore, the downregulation of the immune response through the recruitment of T regulatory cells is important in dictating disease outcomes. Innate receptors such as the toll-like receptors and alarmins have recently been identified in sheep [54, 55] and the suppression of the T regulatory pathways has been described as a mechanism for susceptibility in Scottish Black face sheep infected with* T. circumcincta*  [56].

Thus, given the involvement of the abovementioned cells and mediators identified in development of immunity to GIN parasites, many of these immune parameters have been investigated as selection tools for identification of GIN parasite resistant sheep. However, due to the diverse and complex nature of the immune response, few of these parameters have as yet been substantiated as GIN parasite resistant markers [57, 58].  Table 3 outlines the immune-related selection parameters investigated to date.

#### 2.2.1. Antibodies

Local and peripheral antibody production is associated with GIN parasiteinfection with high levels of parasite-specific IgG_1_, IgE, and IgA correlating with low parasite burden [50, 59]. The role of each antibody isotype in resistance is not fully understood but IgA has been consistently correlated with reduced worm length and fecundity in sheep infected with* T. circumcincta* [60–62]. In* H. contortus* infections, serum IgA and IgG_1_ were consistently higher in genetically resistant sheep compared to randomly bred animals indicating the potential for selection of resistance based on high parasite specific antibody titre [50]. However, the procedures for measuring antibodies in the periphery, while accessible, often give an inaccurate representation of antibody levels (e.g., mucosal) at the infection site [63–66]. Additionally, there is little evidence to support a role for serum antibodies in resistance as opposed to localised antibody production at the site of infection which is more likely to influence the resistance status of an animal [67].

To overcome these issues, other sources of antibodies besides blood have been examined. In faeces, IgG_1_ and IgA can be detected in resistant-bred animals based on low WEC for* H. contortus* [50]. However, false positive readouts due to nonspecific antigen-protein binding terminated development of an assay [68]. The revelation that protective antibodies against larvae potentially derived from the gut associated lymphoid tissue in the intestinal mucosa could be easily detected in the saliva has resulted in the commercialisation of a diagnostic test, the CarLA Saliva Test, for selection of GIN parasite resistant animals [58, 66, 69]. Detection of salivary antibodies is advantageous in comparison to WEC due to earlier detection (at the L3 stage rather than the adult) and saliva from sheep is more appealing for producers to collect than faeces. However to develop an antibody response animals require prior and repeated exposure (not suitable in young animals less than six months which are at most risk) and a certain infection threshold for optimal detection [58], and these limitations may make it impractical for widespread industry adoption.

#### 2.2.2. Eosinophilia

Peripheral blood eosinophilia is associated with GIN parasite infections and has been consistently reported to be higher in sheep resistant to* H. contortus*,* T. circumcincta*, and* T. colubriformis *[70–73]. Such findings led to the evaluation of peripheral blood eosinophilia as a potential marker of GIN parasite resistance. Results correlating blood eosinophilia during* H. contortus* infections with low WEC have been inconsistent (reviewed by [28]), but promising correlations have been found in other GIN parasite infections including* T. colubriformis* and* T. circumcincta* [74]. An early study showed that sheep which responded strongly to vaccination with irradiated* T. colubriformis* had higher blood eosinophilia than low-vaccine responder sheep [71]. This was supported by an extension study which showed that, following vaccination and challenge with the mitogen phytohaemagglutinin, blood eosinophilia was highly correlated to resistance to* T. colubriformis* in random-bred sheep [72]. However, the estimated heritability of blood eosinophilia for selection of resistant sheep was found to be only 43% as effective as using WEC as a selection parameter [75]. Animal behavioural studies have identified that resistant sheep which had increased locomotive patterns also had higher basal circulatory eosinophilia concluding that parasite resistant sheep were also resistant to stress [41]. These results supported those of earlier research in which it was noted that animal handling in cattle generated increased blood basal levels of eosinophils [76]. A more recent study in Scottish Black-faced sheep infected with* T. circumcincta* also found a strong correlation between eosinophilia and resistance and found that the relationship had similar heritability as WEC [60]. However, this relationship with eosinophilia and resistance was age-dependent, existing only in lambs aged 3–7 months [60]. In general, like serum antibodies, the value of peripheral eosinophils as a marker of GIN parasite resistance is confounded by the dynamic nature of the immune system and the changing relationship between the host and parasite interaction [77].

#### 2.2.3. Ghrelin

Ghrelin is a satiety-regulating hormone, stimulating appetite and the release of growth hormones [78]. In sheep, reduced appetite is a symptom of GIN parasitic infection. Recent work has shown that* H. contortus* and* T. colubriformis* resistant and susceptible lines of Merino sheep are divergent in their ghrelin expression [79]. Resistant sheep were observed to have lower basal levels, but following GIN parasite challenge the resistant sheep had higher ghrelin expression (gene and protein) early postinfection than susceptible animals [79]. The function of ghrelin in resistance to GIN parasites is still under investigation but it is believed that the interaction could be direct as ghrelin has previously been shown to have anti-inflammatory properties [80, 81] and immune cells circulating in the blood were found to express ghrelin receptors [79]. A competitive ELISA to detect circulating ghrelin levels has been developed [79] but heritability and the association with increased animal productivity will need to be investigated to order to determine its effectiveness as a marker of GIN parasite resistance.

### 2.3. Diagnostic Based on Inherent Markers

Inherent markers of GIN parasite resistance are categorised as innate markers independent of infection and age of the animal. Consequently, markers based on inherent traits are only useful for breeding of parasite/disease resistant animals. Inherent markers which have been investigated are discussed below.

#### 2.3.1. Blood Type

Early work suggested a link between sheep blood type and resistance to GIN parasites [82]. In sheep, haemoglobin is controlled by two major alleles A and B [9]. Studies have shown that sheep with blood type HbAA are more resistant to* H. contortus* and* T. circumcincta* infections than blood types HbAB and HbBB [82–84]. A similar study investigated blood type as a factor contributing to the responder/nonresponder phenomenon in vaccinated sheep against* T. colubriformis* [85]. Results showed that blood type could not be used to predict if a sheep would respond to vaccination as no associations were found between blood type and WEC during either primary or secondary infections [85]. Further research in this area has not supported the relationship between blood type and resistance to GIN parasites [9].

#### 2.3.2. Immune Cell Markers and Cytokines: Major Histocompatibility Complex and Interferon Gamma

The major histocompatibility complex (MHC) and interferon gamma (IFN-*γ*) genes have had the most attention as candidate genetic markers of GIN parasite resistance. Investigations of polymorphisms within the genes that control MHC class I and II expression stemmed from mice and guinea pig studies in which associations between MHC class II polymorphisms and susceptibility to* Trichinella spiralis* and* T. colubriformis* were identified [86, 87]. Ovine MHC differs from humans and mice in that the MHC class II is only encoded by two genes,* HLA-DQ* and* HLA-DR* [88]. Most attention has been focused on finding associations between* HLA-DR* isoforms and resistance as it is more polymorphic than* HLA-DQ* and is highly expressed on antigen-presenting cells [87]. The translation from rodent models to sheep produced inconsistent results with some researchers finding significant associations between gene variants of MHC and parasite resistance [89–93], whereas other researchers did not [87, 94].

A recent study combining quantitative trait loci (QTL) from cattle, mice, rats, humans, and sheep associated with resistance to internal parasite identified 14 common pathways, four directly involving MHC class II expression [95]. This study also reported the INF-*γ* pathway to be associated with parasite susceptibility supporting earlier genomic work [96–98].

Identifying single gene markers associated with resistance to GIN parasites is difficult as resistance to parasites is considered to be polygenic with hundreds to thousands mutations responsible for the resistant phenotype [99, 100]. However, research continues in the area of genetic markers as they have the advantage over phenotypic markers of measurement prior to birth [87], meaning that producers can make productivity decisions early. Traditionally, application of genetic tools to the selection of animals has been hampered by costs. However, genetic testing for the selection of enhanced animal production traits has now become relatively inexpensive with the development of the ovine single nucleotide polymorphism (SNP) CHIP (OvineSNP50 genotyping BeadChip, Illumina). While the expense of genomic technology has reduced, substantial and continuous investment is essential as large reference and validation animal flocks which closely represent the within and across breed diversity for given traits are required to increase the accuracy of genomic predictions before new genetic traits can enter the industry [101].

#### 2.3.3. Markers of Immunocompetence/Disease Resistant Animals

Breeding for resistance to one infection may result in susceptibility to other pathogens. This statement is based on the theory that natural selection has stabilised intermediate levels of antibody and cell mediated responses to enable an organism to survive against a range of diseases [102]. Consequently, work is now being focused on finding immune traits that give an indication of the overall responsiveness of the immune system. This has been termed immunocompetence.

Recent work in the pork industry has focused on the identification of immunocompetence using traits that are easily measured and heritable [103, 104] and has identified a range of measurable immune traits that are strongly heritable by measuring the type and level of immune cells in blood samples as well as the immune cell's ability to respond to* in vitro *stimulation. Whether these traits can predict disease resistance is still under development.

Cutaneous hypersensitivity reactions are routinely used in humans to determine allergic responses and involve injection of antigens to stimulate a localised inflammatory response. An extension of these studies is whether these inflammatory responses to certain antigens predict the susceptibility or resistant status of animals. In the Canadian dairy industry, researchers have measured delayed hypersensitivity reactions after cutaneous antigen injections to create individual estimated breeding values of cell mediated immunity [105]. These values and estimated breeding values for antibody responses have been correlated to the prevalence of diseases such as mastitis [106]. Additionally, several immune traits measured in the serum have been associated with dairy cattle health in Scotland with higher ratios of CD4+ : CD8+ T lymphocytes associated with reduced occurrences of subclinical mastitis during the lactation period [107].

Cutaneous hypersensitivity reactions have also been investigated in the small livestock industry. An early study examined cutaneous hypersensitivity reactions as a diagnostic for the bacterial infection,* Chlamydia psittaci*, with sheep giving a positive wheal reaction (an increase in eyelid skin thickness), correlating with a decrease in spontaneous lamb abortions [108]. Cutaneous hypersensitivity reactions have been assessed as a potential tool for the identification of sheep resistance to* T. colubriformis *and* H. contortus *infection [72, 109, 110]. Rothwell et al. [72] investigated immune responsiveness in* T. colubriformis*-resistant and susceptible lines of sheep, measuring blood eosinophilia following an intradermal injection of exsheathed L3 (exsheathed L3) and suggest that cutaneous hypersensitivity reactions may be a reliable way to measure the immune system's ability to respond effectively to disease and potentially distinguish between disease resistant and susceptible animals.

Cutaneous hypersensitivity reactions may be a valuable research tool for identifying differential immune responses to various stimuli, due to the relative ease of data collection and sample site monitoring. As research has now implicated an array of immune pathways responsible for resistance to GIN parasites [97, 98], cutaneous hypersensitivity reactions have the potential to explore these mechanisms in more detail in a nonterminal manner and with further development, potential on-farm application. However, limited research to date has focused on cutaneous hypersensitivity reactions to discern parasite resistant animals and the potential of this approach remains unknown. The cost-benefit ratio for producers will also need to be explored and may only be suitable for certain animal production industries.

## 3. Conclusion

Currently there are two primary reasons for use of a diagnostic marker to detect GIN parasites in small ruminants:

1. conserving the effectiveness of anthelmintics,
2. breeding animals with resistance to infection.

Advances in this field have provided a number of diagnostics that are excellent for laboratory-based research with recent molecular advances improving the accuracy and cost-effectiveness of larval identification. However, advances in practical on-farm diagnostics suitable to replace WEC have been limited with many commercialised products being recommended to complement rather than replace WEC. However, a number of immune-based diagnostics show some promise and further understanding of the parasite epidemiology; infection and immune responses of the host will hopefully provide further advancements in the area of practical diagnostics for parasite control in small ruminants.

## Figures and Tables

**Table 1:** Programs/guidelines promoting sustainable GIN parasite control.

Programs	Country	Resource
Wormboss	Australia	http://www.wormboss.com.au/
Wormwise	New Zealand	http://www.beeflambnz.com/farm/tools-resources/wormwise/
Sustainable control of parasites (SCOPS)	United Kingdom	http://www.scops.org.uk/

**Table 2:** Potential and commercial infection-related markers for the diagnosis of GIN parasite infection.

Trait	Description	Advantages	Disadvantages	Application
Worm egg counts (WEC)	Phenotypic, WEC test where the amount of eggs in the faeces is an indicator of adult infection. Eggs counted by microscopy.	(i) Direct measure of infection (ii) On-farm readout (after training)	(i) Labour intensive and involves collection of faeces from the rectum (ii) High variability in counts (influenced by diet, age, degree of infection exposure, hypobiosis, and adult density)	Commercial; http://www.wormboss.com.au/tests-tools/tests/worm-egg-counting.php
	Genotypic, egg counts in faeces detected by DNA analysis.	(i) Direct measure of infection (ii) Quicker than phenotypic WEC (iii) Potentially cheaper as trained experts in worm egg counting are not required	(i) Qualitative only, can't determine infection level and hence resistance status (ii) Involves collection of faeces (iii) Off-farm	Research
	Phenotypic, lectin assay, use of antibodies to detect eggs in faeces.	(i) Direct measure of infection (ii) Can distinguish between species by egg morphology	(i) Involves collection of faeces (ii) Off-farm (iii) Currently only specific only for *H. contortus *	Commercial: http://archive.sheepcrc.org.au/management/worms-flies-lices/ rapid-laboratory-test-for-haemonchus-in-worm-egg-counts.php

Blood loss	Phenotypic, packed cell volume (PCV); involves calculating the percentage of red blood cells from a blood sample.	(i) Direct measurement of infection (ii) Earlier detection of infection compared to WEC	(i) Invasive (ii) Not specific to *H. contortus *infection-other causes of blood loss (iii) Off-farm	Research
	Phenotypic, haemonchus dipstick; blood loss measured by amount detected in faeces.	(i) Quick (ii) No need for highly trained laboratory technician (iii) On-farm	Blood loss is nonspecific therefore recommended to be used in conjunction with WEC	Commercial: http://archive.sheepcrc.org.au/management/ worms-flies-lices/haemonchus-dipstick-test.php
	Phenotypic, FAMACHA^*Ⓒ*^; involves matching eye lid colour with coloured chart indicating level of anaemia.	(i) Noninvasive (ii) Quick (iii) No need for highly trained laboratory technician (iv) On-farm	(i) Nonspecific (other disease induce anaemia) (ii) Subjective measurement	Commercial: http://www.acsrpc.org/Resources/famacha.html

Faecal odour	Phenotypic, infected and uninfected faeces have differential odours detectable by canines.	(i) Detects infection earlier than WEC (ii) Potential for on-farm testing	Can odour be used to determine infection levels? (i) Cost and time to train canines or development of artificial nose?	Research

Animal behaviour	Phenotypic, global positioning systems (GPS) to detect reduced movement found in resistant animals compared to susceptible.	(i) Potential for on-farm testing (ii) Noninvasive	(i) Cost of technology (ii) Indirect measure	Research

Weight loss	Phenotypic, performance based marker which involves calculation of body weight gain based on food efficiency rates.	(i) Potential for on-farm testing (ii) Non-invasive	(i) Cost of technology (ii) Indirect measure	Research HappyFactor

Worm burden	Phenotypic, measuring the number of GIN parasites in the stomach at post mortem.	(i) Distinguish nematode species (ii) Direct measurement of burden (includes exsheathed L3, L4 and adult GIN parasites)	(i) Measurement is terminal (ii) Time consuming (iii) Impractical	Research

Worm weight	Phenotypic, weight of the total amount of GIN parasites collected at post mortem.	Direct measurement of burden (less intensive than counting worm numbers)	(i) Measurement is terminal (ii) Time consuming (iii) Impractical	Research

**Table 3:** Potential and commercialised immune-related markers of GIN parasite resistance.

Trait	Description	Advantages	Disadvantages	Application
Serum antibodies	Phenotypic, enzyme-linked immunosorbent assay (ELISA).	Routine laboratory procedure	(i) Invasive sampling (ii) Off-farm (iii) Not sensitive to infection level (iv) Transient up regulation	Research
Salivary antibodies		(i) Relatively easy collection (ii) Routine laboratory procedure	(i) Off-farm (ii) Requires certain level of infection for detection	Commercialised http://www.kelso.co.nz/partners/ carla%c2%ae-saliva-test-measuring- parasite-immunity-in-sheep/
Faecal antibodies		(i) Relatively easy collection (ii) Routine laboratory procedure	(i) Off-farm (ii) Involves faecal collection (iii) Low accuracy	Research
Blood eosinophilia	Phenotypic, morphological cell differentiation after staining.	Routine laboratory procedure	(i) Invasive sampling (ii) Off-farm (iii) Trained technician required to count eosinophils (iv) Transient up regulation	Research
Ghrelin levels in blood	Phenotypic, ELISA platform. Higher levels in susceptible sheep following infection.	Routine laboratory procedure	(i) Invasive collection (ii) Off-farm (iii) Transient up regulation (iv) Only tested in resistant and susceptible lines	Research
Cutaneous hypersensitivity reactions	Phenotypic, cutaneous injection of sensitised antigen to measure immune function.	(i) Tests responds to a range of diseases (ii) Noninvasive readouts (iii) Potential for on-farm development	(i) Involves injection of antigens into animals (ii) 2–24 hr time delay for readout	Research
